# Cerium oxide nanoparticles for enhanced healing of diabetic foot ulcers

**DOI:** 10.3389/fbioe.2025.1695821

**Published:** 2026-01-12

**Authors:** Ziyue Fu, Ling Chen, Lingjiang Mo, Yuefu Zhan, Liheng Ma

**Affiliations:** 1 The First Affiliated Hospital of Guangdong Pharmaceutical University, Guangzhou, China; 2 The Third People’s Hospital of Longgang District Shenzhen, Shenzhen, China

**Keywords:** cerium oxide nanoparticles, diabetic foot ulcers, coating strategies, antioxidant activity, anti-inflammatory, biocompatibility, angiogenesis, extracellular matrix remodeling

## Abstract

One of the main complications of diabetes is diabetic foot ulcers (DFUs), which have a substantial global impact on morbidity, medical expenses, and lower limb amputations. A complicated microenvironment—characterized by bacterial infections, chronic inflammation, oxidative stress, and poor angiogenesis impedes the healing process of DFUs. Cerium oxide nanoparticles (CeO_2_ nanoparticles), owing to their antibacterial, anti-inflammatory, and antioxidant properties, have emerged as a promising treatment. These nanoparticles can scavenge reactive oxygen species (ROS), reduce inflammation, and stimulate tissue regeneration by modulating macrophage polarization and promoting angiogenesis. However, limitations such as poor biocompatibility, aggregation, and dose-dependent toxicity hinder their clinical application. Recent advances in coating strategies—including polymeric, natural biomolecule, and hybrid coatings have improved the stability, targeting, and controlled release of CeO_2_ nanoparticles. This review discusses the design, therapeutic mechanisms, and safety considerations of coated CeO_2_ nanoparticles in DFU treatment, with emphasis on combination therapies and smart-responsive coatings. Clinical translation challenges and future research directions are also addressed.

## 1 Introduction

Diabetic wounds, often associated with diabetes mellitus, represent a complex metabolic disorder and a major global public health concern. These wounds significantly impair quality of life and are a leading cause of disability. Wound healing is a dynamic and multifaceted process involving stages of inflammation, proliferation, and remodeling. The burden of diabetic wounds on the healthcare system is immense, with global wound care costs estimated to reach tens of billions of dollars annually (Hao et al., 2024; Chen et al., 2024). The annual incidence of DFUs is approximately 2.2%, while the lifetime risk exceeds 30%. Even after healing, the recurrence rate within 1 year is nearly 40%. What is more, DFUs are implicated in 80% of lower limb amputations and have a 5-year mortality rate of 50%–70% (Waibel et al., 2023; Armstrong et al., 2023; McDermott et al., 2023).

There is a wide range of treatments available for the healing of both acute and chronic wounds. Standard clinical treatments for wounds encompass various approaches such as skin perfusion recovery, infection management, metabolic control, complication treatment, local wound care, debridement, administration of growth factors, pressure regulation, hyperbaric oxygen therapy, and physical therapy (Chen et al., 2024). Immediate treatment, such as intravenous antibiotics and fluid resuscitation, should be administered promptly to prevent further deterioration. Antibiotics act by directly disrupting the cell envelope and lethally destroying genetic traits, leading to irreversible loss of unique characteristics of specific bacterial strains and inhibiting replication of both Gram-positive and Gram-negative bacteria. Nevertheless, inappropriate and indiscriminate use of antibiotics has contributed to the evolution of exogenous or endogenous drug-resistant strains (Hon et al., 2024; Li et al., 2023). Debridement, which may cause pain and bleeding and require trained personnel, eliminates necrotic tissue and contaminated material to encourage the regeneration of healthy tissue. Revascularization is a riskier and more expensive procedure that involves angioplasty, stenting, and bypass surgery to increase blood flow to the lower limbs (Nosrati et al., 2023). Although decompression is fundamental, it can lead to significant limitations in daily life, mainly due to the resulting reduction in physical activity (Waibel et al., 2023). When it comes to DFU, autologous skin grafts are the preferred and most effective treatment. The procedure entails grafting the harvested tissue to the incision after removing the patient’s healthy skin area’*s epidermis* and a portion of the underlying dermis. However, wounds that cover more than 60% of the patient’s body surface area are not covered by it. Severe scarring may result from inadequate and delayed skin regeneration at the tissue harvest site. Other issues in the donor area include soreness, pigmentation issues, and issues with hair regeneration (Nosrati et al., 2023). Conventional dressings such as dry gauze provide an isolation barrier, but are not antimicrobial and may cause a secondary injury (Jiang et al., 2023). CeO2 NPs are recognized for their capabilities in enhancing wound closure, minimizing scarring, mitigating inflammation, and exerting antibacterial effects, which have led to their prominence in wound care research (Table 1) (Chen et al., 2024).

**TABLE 1:** Comparison of therapeutic characteristics between cerium oxide nanoparticles (CeO_2_ NPs) and traditional therapies for diabetic foot ulcer treatment.

Indicator	CeO_2_ NPs	Traditional therapy (e.g., PDGF Gel)
Antioxidant capacity	Actively scavenges ROS	Relies on exogenous antioxidants (e.g., Vitamin E)
Efficacy duration	Long-lasting (controlled release design)	Short-term (requires frequent administration)
Cost-effectiveness	High (potential for large-scale production)	Very high (growth factor preparations)
Safety	Local toxicity controllable, systemic risks need verification	Low toxicity but limited efficacy

Cerium oxide nanoparticles exhibit a strong antioxidant capacity through valence conversion between Ce^3+^/Ce^4+^ and the presence of oxygen vacancies. This redox activity enables CeOx to scavenge reactive oxygen species (ROS), particularly superoxide anion (-O_2_-) and hydrogen peroxide (H_2_O_2_), via electron donation or acceptance (Zhu et al., 2024). In addition, on account of their antioxidant properties, CeO_2_ NPs can reduce the accumulation of intracellular ROS, thereby indirectly decreasing oxidative stress-induced inflammatory signaling (Yi et al., 2024). Cerium oxide nanoparticles effectively inhibit the inflammatory response of endothelial cells in a highly oxidized microenvironment by inhibiting the NLRP3 inflammatory vesicle pathway, regulating gene expression, and influencing signaling and immune-related pathways (He et al., 2024). In combination with antibiotics, it can reduce the dose and enhance biofilm penetration (Wang et al., 2021). However, the current application of CeO_2_ NPs also has limitations biocompatibility deficiency. Unmodified NPs are prone to aggregation, leading to liver/spleen accumulation and long-term toxicity, with 10 nm NPs causing liver damage in mice (Casals et al., 2020).

Single CeO_2_ NPs lack responsiveness to the trauma microenvironment (e.g., pH, ROS), which leads to sudden drug release or premature inactivation (Zhao C. et al., 2024; Yuan et al., 2022; Kamalipooya et al., 2024). Moreover, there are constraints in clinical translation, difficulties in achieving large-scale production, and a lack of uniform safety standards. The single-use nature of the majority of CeO_2_ NPs is overcome by the development of coating technology. Surface changes (e.g., PEG, chitosan) give NPs more stability, targeting, and functional variety (Yadav et al., 2023). This review aims to provide a systematic overview of the material design and function of coated CeO_2_ NPs, clarify the molecular mechanisms through which they target and regulate the DFU microenvironment, thoroughly evaluate the safety issues and risk mitigation strategies, and suggest future research avenues, including combination therapies and smart-responsive coatings.

## 2 DFU wound microenvironment

**FIGURE 1:**
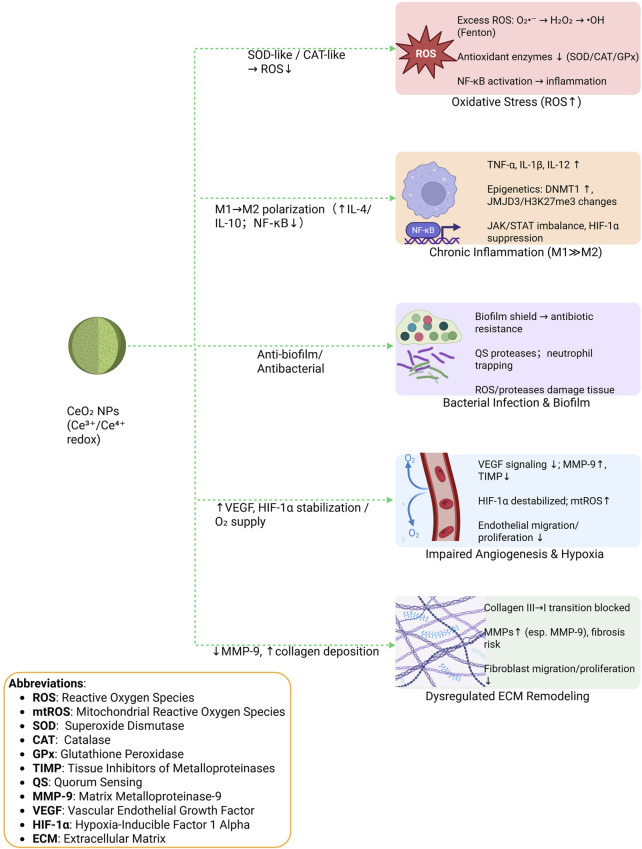
Diabetic foot ulcer microenvironment and multi-target actions of CeO_2_ nanoparticles.

Numerous pathogenic processes, including oxidative stress imbalance, chronic inflammation, bacterial infection, poor angiogenesis, and extracellular matrix (ECM) dysregulation, are involved in the complex trabecular milieu of diabetic foot ulcers (DFUs). By altering these microenvironmental elements, cerium oxide nanoparticles (CeO_2_ nanoparticles) have become a viable therapy option for DFU (Figure 1).

### 2.1 Oxidative stress and ROS accumulation

ROS are generated during normal metabolic processes and play a pivotal role in many antibacterial responses. The ROS cascade involves the sequential production of superoxide anion radicals, hydrogen peroxide (H_2_O_2_), and hydroxyl radicals, regulated by enzyme complexes such as nicotinamide adenine dinucleotide phosphate (NADPH) oxidase and individual enzymes including superoxide dismutase (SOD). In the presence of iron ions, the Fenton reaction produces highly reactive hydroxyl radicals from H_2_O_2_. These ROS mediate immune responses by recruiting lymphocytes, regulating local blood flow, promoting neovascularization at wound sites, and transmitting signals to both immune and non-immune cells. Inflammatory processes further amplify ROS production; for instance, activated neutrophils and macrophages generate ROS as part of bacterial clearance.

Dysregulation of insulin—either deficiency or resistance—leads to elevated blood glucose levels. Under normal conditions, glucose homeostasis is maintained by hormonal and neural mechanisms; however, in diabetic states, hyperglycemia activates mitochondrial respiratory pathways, including the sorbitol and polyol pathways, resulting in excessive ROS production. The resulting oxidative stress disrupts intracellular redox balance, contributing to cellular dysfunction.

Normally, excess ROS are detoxified by intracellular antioxidant enzymes such as glutathione peroxidase (GPx), catalase (CAT), and SOD. In diabetic wounds, hyperglycemia, chronic inflammation, and other pathological factors impair the activity of these enzymes, limiting ROS scavenging. Excess ROS can damage proteins, DNA, and cell membranes, leading to cellular malfunction or death. Moreover, ROS can activate pro-inflammatory signaling pathways, such as nuclear factor kappa B (NF-κB), further amplifying inflammation and establishing a vicious cycle.

CeO_2_ nanoparticles mimic the activity of antioxidant enzymes, including SOD, CAT, and peroxidase (POD). SOD-like activity converts superoxide anions into hydrogen peroxide, which is subsequently decomposed into water and oxygen via CAT-like activity. Through these mechanisms, CeO_2_ nanoparticles effectively reduce ROS levels and oxidative stress, creating a more favorable microenvironment for diabetic wound healing (Chen et al., 2024; Li et al., 2023; Nosrati et al., 2023).

### 2.2 Chronic inflammation and macrophage polarization

Diabetic wounds frequently fail to progress through the reparative phase due to sustained chronic inflammation. Although diabetes generally promotes persistent inflammatory responses, controlled acute inflammation—mediated by cytokines such as interferon-gamma (IFN-γ)—is crucial for effective tissue repair and regeneration (Theocharidis et al., 2020). Macrophages play dual roles in wound healing, exhibiting pro-inflammatory (M1) or anti-inflammatory/restorative (M2) phenotypes. In normal healing, macrophages transition from the M1 to M2 state; however, in diabetic wounds, this polarization shift is delayed, resulting in prolonged M1 dominance and excessive secretion of pro-inflammatory cytokines, including tumor necrosis factor-alpha (TNF-α) and interleukin-12 (IL-12).

M1 macrophages rely on rapid glycolysis to meet high ATP and biosynthetic demands, fueling the pentose phosphate pathway, ROS generation, and synthesis of amino acids, nucleotides, and NADPH. Metabolic intermediates such as acetyl-CoA and succinate further coordinate inflammatory signaling, stabilizing HIF-1α and IL-1β to regulate cellular responses under hypoxic conditions (Monaghan et al., 2023). Hyperglycemia also disrupts epigenetic regulation: overproduction of histone demethylases (e.g., JMJD3) and high DNMT1 levels suppress M2 polarization, while interventions such as DNMT1 inhibitors or modulation of MLL1-mediated H3K4me3 can restore M2 macrophage function. Advanced glycation end-products (AGEs) further reinforce M1 polarization via the RAGE/NF-κB pathway, sustaining a pro-inflammatory microenvironment. Disruptions in JAK/STAT signaling additionally impair M2 polarization.

Epidermal stem cells (ESCs) can promote M2 polarization and accelerate healing by stimulating fibroblast and macrophage growth (Deng et al., 2023). Natural antioxidants, such as Haematococcus pluvialis extract (HEA) containing astaxanthin (AST), have also been reported to reduce M1 macrophages, increase M2 macrophages, scavenge ROS, and suppress pro-inflammatory cytokines (Kang et al., 2024). While the above studies demonstrate the effects of antioxidants on macrophage polarization, the distinct role of CeO_2_ nanoparticles in modulating macrophage behavior deserves dedicated attention.

CeO_2_ NPs possess a redox-active surface (Ce^3+^/Ce^4+^) that enables intrinsic catalytic activity, allowing them to modulate the local oxidative microenvironment and influence immune responses. In principle, this activity could promote M2 polarization and enhance anti-inflammatory cytokine expression (e.g., IL-4, IL-10), while reducing pro-inflammatory cytokines (Le et al., 2025; Zhao S. et al., 2024). Nevertheless, direct evidence demonstrating that CeO_2_ NPs alone can induce M2 polarization is still limited. In many experimental systems, additional factors—such as miRNA delivery or biomolecular coatings—may contribute synergistically to macrophage modulation (Bejerano et al., 2018). Distinguishing the specific role of CeO_2_ from these combined approaches is essential for clarifying its mechanistic effect and optimizing therapeutic design.

### 2.3 Bacterial infection and biofilm formation

In DFUs, pathogenic bacteria establish cooperative interactions that create a microenvironment conducive to chronic wound persistence and infection spread. Chronic wounds provide an ideal niche for polymicrobial sessile communities (PSCs), with *Pseudomonas aeruginosa* and *Staphylococcus aureus* among the most prevalent pathogens. These bacteria form biofilms—dense, self-produced polymeric matrices on the wound surface—that protect them from antibiotics and host immune responses, resulting in high resistance to antimicrobial agents. The accumulation of necrotic tissue and wound debris further facilitates bacterial adhesion and biofilm maturation. Unlike planktonic bacteria, biofilms are notoriously difficult to eradicate, representing a major challenge for conventional antimicrobial therapies.

The host immune system mounts a persistent inflammatory response against biofilms, recruiting neutrophils and macrophages. These immune cells release large quantities of ROS and proteases, which not only damage regenerating tissues but also degrade components of the immune system, thereby delaying wound healing. Additionally, quorum-sensing (QS)–regulated proteases, particularly from *P. aeruginosa*, promote immune evasion by clustering neutrophils near the biofilm, preventing them from penetrating and eliminating the embedded bacteria. While planktonic bacteria can trigger macrophage-mediated release of pro-inflammatory cytokines such as TNF-α and interleukin-6 (IL-6), this defense mechanism is significantly impaired in the presence of biofilm matrices.

Bacterial infections act synergistically with AGEs, further impairing host immunity and exacerbating chronic inflammation. This sustained inflammatory microenvironment promotes biofilm development, reduces drug penetration, and perpetuates non-healing wounds (Afonso et al., 2021; Zhang et al., 2022).

Emerging therapeutic strategies for combating biofilms include antimicrobial peptides (AMPs), photothermal therapy (PTT), photodynamic therapy (PDT), polymer-based catalyst delivery systems (Polyzymes), polymer-based drug delivery systems, and functional wound dressings (Li et al., 2023). Although preclinical and laboratory studies have shown promising results, challenges remain regarding stability, safety, cost, and clinical adaptability. Future research should focus on refining existing technologies, developing novel materials and strategies, and leveraging personalized medicine approaches to enhance the efficacy and safety of anti-biofilm therapies.

### 2.4 Impaired angiogenesis and hypoxia

Chronic inflammation and oxidative stress in diabetic wounds disrupt endothelial cell function, reduce responsiveness to growth factors such as VEGF, and impair angiogenesis. Elevated pro-inflammatory cytokines (e.g., IL-6, TNF-α) and dysregulated matrix metalloproteinases (particularly MMP-9) degrade extracellular matrix components and interfere with endothelial signaling, while reduced TIMP levels further exacerbate vascular dysfunction. Consequently, neovascularization is insufficient, contributing to poor wound healing in DFUs (Nosrati et al., 2023).

Hypoxia is another hallmark of diabetic wounds, driven by reduced perfusion due to peripheral vascular disease and hyperglycemia-induced mitochondrial dysfunction. Oxygen deprivation impairs fibroblast proliferation and collagen production, creating a feedback loop in which hypoxia further enhances inflammation. HIF-1α, a key regulator of hypoxia response and angiogenesis, exhibits reduced transcriptional activity and protein stability in diabetic wounds due to PHD-dependent and independent degradation mechanisms. This leads to diminished VEGF expression, reduced fibroblast migration, and impaired angiogenesis (Song et al., 2024; Catrina and Zheng, 2021).

Multifunctional CeO_2_ nanoparticles, such as Mesoporous Silica Nanoparticle loaded with Metformin and Cerium Dioxide (MSN@Met-CeO_2_), can directly target microenvironmental dysfunctions in diabetic wounds. By scavenging both mitochondrial and extracellular ROS through redox and catalase-like activity, these nanoparticles reduce oxidative stress, alleviate hypoxia, and restore conditions favorable for endothelial cell function and angiogenesis. In addition to ROS clearance, CeO_2_ NPs locally release oxygen, further enhancing tissue oxygenation and creating an environment conducive to vascular regeneration.

Mechanistically, MSN@Met-CeO_2_ further reinforces its therapeutic effects by modulating intracellular signaling. Activation of the AMP-activated protein kinase (AMPK) pathway induces the phosphorylation of Yes-associated protein (YAP), which restricts its nuclear translocation and downregulates the expression of dynamin-related protein 1 (DRP1). This reduction in DRP1 decreases mitochondrial fission, thereby lowering mitochondrial ROS production. By coordinating the regulation of mitochondrial dynamics with extracellular ROS scavenging, MSN@Met-CeO_2_ facilitates endothelial cell recovery, promotes angiogenesis, and accelerates wound healing under diabetic conditions (Li et al., 2024).

Additionally, while localized oxygen therapies, such as hyperbaric oxygen treatment, can alleviate hypoxia, their high cost and variable efficacy pose limitations (Afonso et al., 2021). In this context, the intrinsic antioxidant activity of CeO_2_ NPs may act synergistically with oxygen therapy to optimize tissue oxygenation and enhance wound repair.

#### 2.4.1 Permission to reuse and copyright

Permission must be obtained for use of copyrighted material from other sources (including the web). Please note that it is compulsory to follow figure instructions.

### 2.5 Dysregulated extracellular matrix (ECM) remodeling

Aberrant ECM remodeling is a major contributor to delayed healing in diabetic wounds. Chronic hyperglycemia impairs fibroblast function by reducing cell migration and proliferation while increasing apoptosis, thereby limiting their differentiation into myofibroblasts—cells essential for ECM deposition and contraction. In diabetic models, myofibroblast populations are significantly reduced during early healing phases. This dysfunction is associated with disrupted cytokine signaling (e.g., TGF-β), enhanced oxidative stress, and abnormal fibroblast activity, ultimately leading to disordered ECM deposition and tissue fibrosis (Liu et al., 2022).

Moreover, hyperglycemia stimulates pro-inflammatory cytokines (e.g., TNF-α, IL-1β), growth factors (e.g., PDGF, TGF-β), and accumulation of AGEs, all of which dysregulate collagen and elastin synthesis. Overexpression of MMPs, particularly in chronic wounds, exacerbates ECM degradation and prevents normal matrix remodeling, including the physiological transition from collagen type III to type I.

Recent studies also underscore the role of epigenetic regulation in ECM dysfunction. Upregulation of JMJD3 promotes pro-inflammatory gene expression and suppresses ECM-related pathways. Concurrently, elevated DNMT1 activity and histone methylation imbalances (e.g., reduced H3K4me3 and increased H3K27me3) hinder anti-inflammatory gene activation and macrophage polarization, further impairing the healing microenvironment (Deng et al., 2023). Cell behavior in diabetic wounds, including migration, proliferation, differentiation, and ECM synthesis, is impacted by malfunctioningECM components. (Xiang et al., 2024).

To address these challenges, biomimetic materials have emerged as promising therapeutic tools. Kamalipooya et al. developed PCL/CA/CeO_2_–CSNPs electrospun nanofibers, which structurally mimic native ECM and enhance fibroblast adhesion, migration, and proliferation. The embedded CeO_2_–chitosan nanoparticles provide potent antioxidant and antibacterial activity, mitigating oxidative stress and infection while promoting ECM repair. These nanofibers significantly improved cell survival (∼90.3% at 48 h) and supported tissue regeneration under diabetic conditions (Kamalipooya et al., 2024).

In summary, targeting ECM remodeling through epigenetic modulation and biomimetic nanostructures may provide a synergistic strategy for restoring structural and functional integrity in diabetic wounds.

## 3 Coated CeO_2_ NPs: from material design to therapeutic breakthroughs

### 3.1 Design and synthesis strategies for coating materials

Surface modification of CeO_2_ nanoparticles (CeO_2_ NPs) with polymers, biomolecules, or hybrid coatings enhances their stability, biocompatibility, and therapeutic efficacy. Polymer coatings (e.g., PEG, PLGA) improve dispersion and preserve enzyme-like activity, while biomolecule coatings (e.g., gelatin, chitosan, dextran, HA) support cell adhesion, neovascularization, and controlled release. Hybrid coatings (e.g., mesoporous silica) further reduce degradation and enable sustained therapeutic effects. CeO_2_ NPs can be synthesized via chemical, physical, or green methods, which determine particle size, morphology, and redox properties, ultimately influencing biological activity. Optimizing coating type and synthesis strategy is key for translating CeO_2_ NPs into biomedical applications, including wound healing and regenerative therapies.

#### 3.1.1 Selection of coating type

**FIGURE 2:**
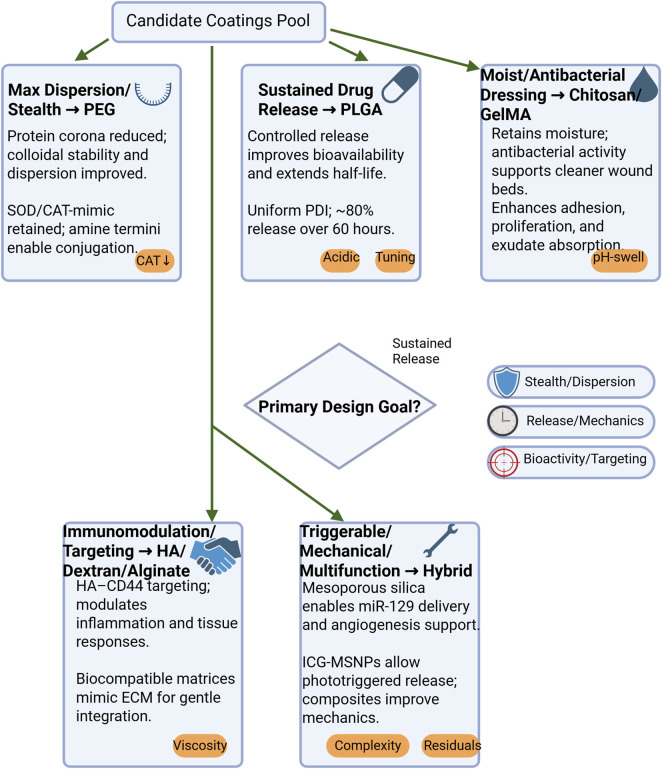
Decision tree for material/coating selection and associated risks.

Cerium oxide nanoparticles (CeO_2_ NPs) can be synthesized through various methods, including chemical, physical, and green synthesis approaches. Each method offers distinct advantages in controlling the size, morphology, and functional properties of the nanoparticles, making them suitable for specific applications (Chen et al., 2024) (Figure 2).

Chemical methods, such as co-precipitation, hydrothermal synthesis, sol-gel reactions, and thermal decomposition, provide precise control over particle size, surface properties, and the Ce^3+^/Ce^4+^ ratio, which are crucial for optimizing catalytic and antioxidant activities. For instance, hydrolysis of cerium nitrate under mildly alkaline conditions produces CeO_2_ NPs with an average size of ∼5.4 nm. Adjusting the pH during synthesis can further enhance the antioxidant capacity of the nanoparticles, while heat treatment improves colloidal stability and homogeneity (Guerra-Ojeda et al., 2022; Shlapa et al., 2022).

Surfactants, such as cetyltrimethylammonium bromide (CTAB) and CTMAB, can modulate the morphology of CeO_2_ NPs by promoting anisotropic crystal growth (e.g., rods or dumbbells) (Dai et al., 2022). This morphology enhancement increases the nanoparticles’ ability to scavenge reactive oxygen species (ROS) and, when combined with AgBr nanoparticles, further enhances antimicrobial properties (Zhang et al., 2021). In addition, Karunakaran et al. demonstrated the use of CTAB-assisted synthesis to produce Zn-doped hydroxyapatite nanorods with improved size control, antibacterial efficacy, and biocompatibility, as confirmed through zebrafish toxicity assays (Karunakaran et al., 2023).

Citric acid, another chelating agent, plays a key role in preventing particle aggregation during synthesis. By adjusting valence states, citric acid enhances the dispersion, crystallinity, and catalytic performance of CeO_2_ NPs, ensuring better stability and efficiency in various applications (Collin et al., 2022; Barrios et al., 2017).

Bakare et al. further optimized the sol-gel method by using a combination of low-concentration CTAB and citric acid. CTAB facilitated the formation of mesoporous structures with defined grain boundaries, while citric acid prevented premature metal ion precipitation, improving particle stability. The resulting CeO_2_ NPs exhibited a high surface area and enhanced biodegradability, making them suitable for biomedical applications (Bakare et al., 2025).

Physical approaches, such as ball milling, provide a straightforward route to reduce particle size (∼4.5–20 nm) without toxic reagents, but can lead to irregular shapes, aggregation, and require extensive post-processing (Gomaa et al., 2023).

In recent years, green synthesis methods have attracted considerable attention due to their environmental friendliness and inherent biocompatibility. These strategies employ plant, fungal, polymeric, or nutrient extracts as reducing and stabilizing agents (Chen et al., 2024). CeO_2_ nanoparticles produced via green synthesis exhibit favorable properties, including strong antibacterial activity and high cytocompatibility, making them well-suited for diverse biomedical applications. For example, Kochiae Fructus extract was used to fabricate KF-CeO_2_ NPs through a low-temperature process, yielding nanoparticles (∼11.3 ± 3.9 nm) that demonstrated potent antibacterial effects (>95% inhibition of multidrug-resistant pathogens such as *K. pneumoniae* and *S. aureus*) and high cytocompatibility with human mesenchymal stem cells and mouse fibroblasts (Lu et al., 2022).

Although most studies have examined CeO_2_ NP interactions with mesenchymal stem cells (MSCs), their effects on endothelial progenitor cells (EPCs) and endothelial colony-forming cells (ECFCs)—critical regulators of angiogenesis and vascular repair in chronic wounds like diabetic foot ulcers (DFUs)—remain largely unexplored (Huang et al., 2025). Investigating CeO_2_ NP interactions with EPCs/ECFCs represents a promising avenue to enhance vascular regeneration and therapeutic outcomes, linking nanoparticle design directly with functional biological applications.

Furthermore, the choice of synthesis method and the specific plant or biomolecular extract employed can significantly influence the physicochemical characteristics, crystallinity, and biocompatibility of CeO_2_ NPs, thereby modulating their biological performance. For instance, extracts from Viscum album and Chelidonium majus produced highly pure CeO_2_ NPs with controlled lattice parameters (∼5.376–5.398 Å) and high crystallinity (Fifere et al., 2023). In a similar approach, Kamalipooya et al. utilized Thymus vulgaris extract to synthesize CeO_2_ NPs, which were subsequently encapsulated in chitosan nanoparticles. This hybrid system exhibited strong antioxidant activity (up to 88.27%) and enhanced wound healing potential, attributable to the polyphenols and flavonoids present in the extract (Kamalipooya et al., 2024).

Despite these advantages, green synthesis methods face challenges such as low product yield and the need for large volumes of organic extracts. Future efforts should therefore focus on scaling up green synthesis through process simplification, cost reduction, and integration with biomedical technologies, including drug delivery and wound therapy platforms (Nadeem et al., 2020). Optimizing synthesis strategies in this way will be critical for translating multifunctional CeO_2_ NPs from laboratory research to clinical and industrial applications.

#### 3.1.2 Preparation process optimization

**FIGURE 3:**
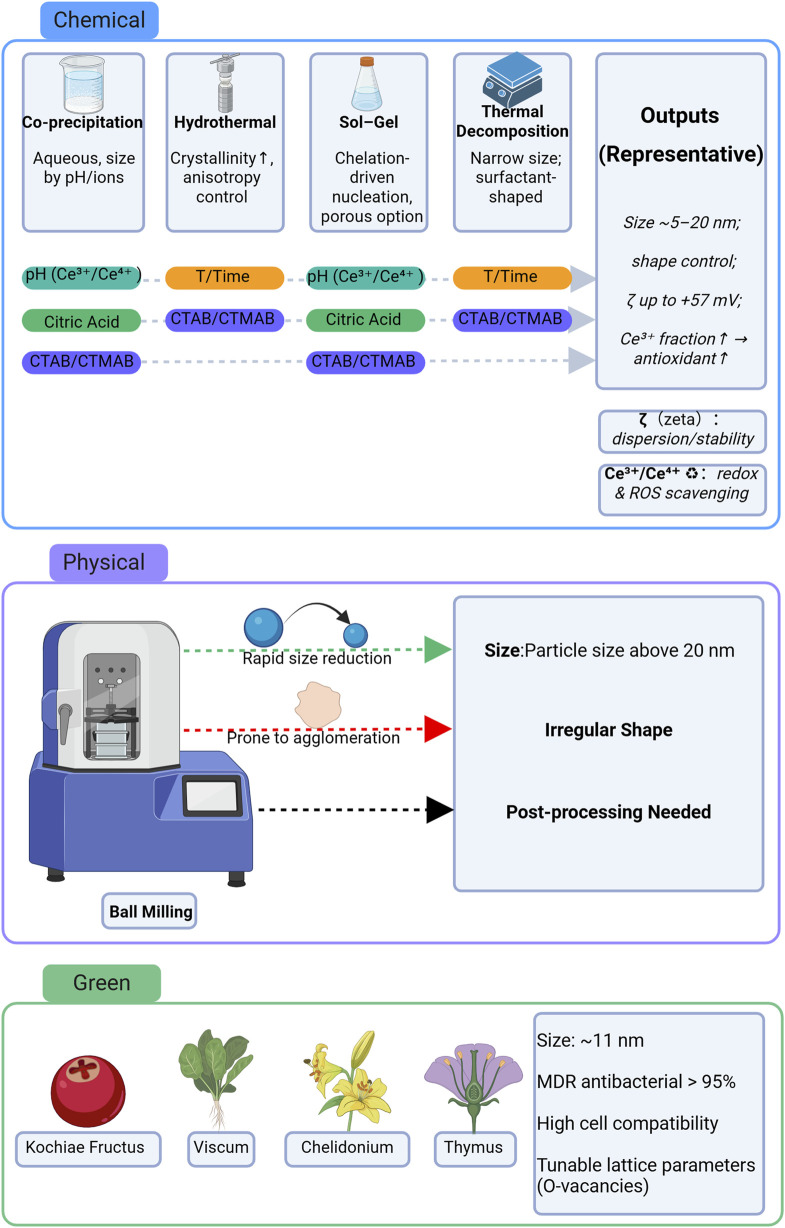
Overview of chemical, physical, and green synthesis methods of CeO_2_ nanoparticles with key parameters and resulting properties.

Surface coatings are critical for improving nanoparticle stability, biocompatibility, and functionality, and can be classified into polymer, natural biomolecule, and hybrid inorganic-organic coatings (Figure 3).

Polymer-based coatings, such as poly (ethylene glycol) (PEG) and poly (lactic-co-glycolic acid) (PLGA), are widely used to enhance the dispersion, biocompatibility, and stability of CeO_2_ nanoparticles (NPs). PEG coatings, in particular, improve colloidal stability by forming a brush-like structure on the nanoparticle surface, reducing protein adsorption and preventing aggregation. According to Goujon et al., PEGylation not only increases dispersion but also significantly enhances biocompatibility by minimizing immune recognition. Notably, PEG-coated CeO_2_ NPs retain their superoxide dismutase (SOD)-like and catalase (CAT)-like enzyme mimetic activities, although multi-PEG coatings slightly reduce CAT-like activity by approximately 20% (Goujon et al., 2021).

However, the influence of surface coatings on the bioactivity of CeO_2_ NPs, especially regarding their intrinsic redox activity, requires careful consideration. CeO_2_ NPs are known for their pseudo-enzymatic activity due to the Ce^3+^/Ce^4+^ redox cycle on the surface (Kurlov et al., 2016). Coating CeO_2_ NPs with polymers may interfere with these active centers, potentially reducing their antioxidant and enzymatic activities. While PEGylation improves stability and biocompatibility, it may inhibit the redox catalytic processes critical for CeO_2_’s intrinsic bioactivity. To better understand how various coatings affect CeO_2_ NP bioactivity, a comparative analysis of key activities—such as SOD-like and CAT-like activities—would provide valuable insights into the functional modulation of CeO_2_ NPs, helping to optimize their biomedical applications. This analysis is presented in Table 2.

**TABLE 2:** Effects of different surface coatings on the enzyme-mimetic activities, Ce^3+^/Ce^4+^ redox cycling, and bioactivity of cerium oxide nanoparticles (CeO_2_ NPs).

Coating type	SOD-like activity	CAT-like activity	Effect on Ce^3+^/Ce^4+^ redox cycle	Bioactivity changes	References
Uncoated CeO_2_ NPs	High	Moderate	Uninterrupted Ce^3+^/Ce^4+^ redox cycle	High catalytic activity, potential cytotoxicity at high doses	Kurlov et al. (2016)
PEG-coated CeO_2_ NPs	Moderate	Reduced (∼20%)	Reduces Ce^3+^/Ce^4+^ activity	Improved stability and biocompatibility, but slight reduction in redox activity	Goujon et al. (2021)
PLGA-coated CeO_2_ NPs	Moderate	Moderate	Slight reduction in redox cycle	Enhanced drug delivery, stability in physiological conditions	Pridgen et al. (2013)
CTAB-coated CeO_2_ NPs Dextran-coated CeO_2_ NPs	High High	High High	Enhances Ce^3+^/Ce^4+^ redox cycle Preserves Ce^3+^/Ce^4+^ balance	Improved ROS scavenging and antibacterial properties Improved biocompatibility and cellular interactions	Dai et al. (2022) (Andrabi et al., 2020)

In this context, Ren et al. demonstrated that PEG-coated CeO_2_ NPs, when introduced into human umbilical cord mesenchymal stem cells, exhibited potent antioxidant activity across several assays, including SOD/CAT mimetic evaluation, DPPH radical scavenging, and hydroxyl radical neutralization. These NPs effectively reduced superoxide and H_2_O_2_ production in a dose-dependent manner, supporting their utility in mitigating oxidative stress (Ren et al., 2023). Similarly, Qi et al. reported that CeO_2_ NPs modified with phosphorylated oligomers exhibited superior redispersibility in various organic solvents, including acetic acid, ethanol, and chloroform, enhancing their versatility for biomedical applications (Qi et al., 2008).

Beyond PEG, PLGA coatings also play a significant role in improving the functionality of CeO_2_ NPs. PLGA, a synthetic copolymer composed of lactic acid and glycolic acid units, is extensively used in drug delivery systems for its controlled-release capabilities and biodegradability. Studies have shown that PLGA-coated CeO_2_ NPs significantly improve drug stability and bioavailability. For instance, in diabetic mice, PLGA-based formulations enhanced cardiac function and reduced inflammatory cytokine levels, indicating improved therapeutic performance (Pridgen et al., 2013). Gourishetti et al. demonstrated that silymarin-loaded PLGA nanoparticles maintained uniform morphology (PDI <0.200), sustained drug release (∼80% over 60 h), and exhibited an extended half-life (∼13.95 h), confirming their slow-release potential (Gourishetti et al., 2020).

Natural biomolecule coatings—including gelatin, chitosan, dextran, sodium alginate, and hyaluronic acid (HA)—impart inherent biocompatibility and functional bioactivity. GelMA and dextran–curcumin hydrogels, for example, improve fibroblast and keratinocyte survival, retain moisture, and promote angiogenesis (Xue et al., 2024; Augustine et al., 2021; Andrabi et al., 2020). HA-based coatings can modulate inflammation and protect tissue architecture (Jin et al., 2024).

Hybrid coatings combine inorganic and organic materials to address degradation and residue accumulation. Zhou et al. synthesized mesoporous silica–CeO_2_ nanocomplexes (MS-CeO_2_) loaded with microRNA-129 (miR-129), which exhibited excellent radioprotective and regenerative effects *in vitro* and *in vivo* through enhanced proliferation, angiogenesis, and anti-apoptotic activity (Zhou D. et al., 2022). Additionally, photosensitizer-triggered release systems, such as indocyanine green (ICG)-activated mesoporous silica nanoparticles (MSNPs), have demonstrated controllable therapeutic release profiles (Kolanthai et al., 2022).

Further, Multifunctional scaffolds, such as chitosan–CeO_2_/TiO_2_/PCL constructs, integrate mechanical strength, antimicrobial activity, and controlled exudate absorption, highlighting the broad therapeutic potential of engineered coatings (Kamalipooya et al., 2024).

All in all, surface modification of CeO_2_ NPs with diverse coating strategies significantly enhances their stability, biocompatibility, and therapeutic applicability. Each coating type—polymer, biomolecule, or hybrid—offers distinct advantages and challenges, and the ongoing optimization of these materials will be pivotal for future clinical translation in wound healing and related regenerative therapies.

### 3.2 Comprehensive treatment of infected ulcers

Building on the design and coating strategies described above, CeO_2_ NPs can be integrated into therapeutic platforms targeting the multifactorial defects of diabetic foot ulcers (DFUs), including oxidative stress, bacterial infection, and chronic inflammation. Key representative preclinical studies summarizing CeO_2_ NP formulations, surface modifications, biological models, and their main therapeutic outcomes are summarized in Table 3, providing a clear overview of their effects on oxidative stress, inflammation, angiogenesis, and macrophage modulation.

**TABLE 3:** Representative preclinical studies of CeO_2_ nanoparticles (NPs) in diabetic wound healing.

Nanoparticle formulation	Surface modification	Biological model	Main biological outcomes	Macrophage/Cytokine effects	References
A. *In vitro* studies					
PEG-CeO_2_	PEGylation	Human MSCs	Antioxidant, Anti-inflammatory	M1↓, M2↑, IL-6↓, IL-10↑	Goujon et al. (2021)
KF-CeO_2_	Kochiae Fructus extract	MSCs, fibroblasts	Antioxidant, Antibacterial	M2↑, TNF-α↓, IL-1β↓	Lu et al. (2022)
CNP-miR146a	miR146a conjugation	Cultured fibroblasts	Antioxidant, Angiogenesis	NF-κB↓, IL-6↓, IL-8↓	Zhou et al. (2022a)
PCL/CA/CeO_2_–CSNPs nanofibers	PCL + Citric Acid + CeO_2_–Chitosan	Fibroblasts	Antioxidant, Antibacterial, ECM repair, Cell survival ↑ (∼90.3% at 48 h)	M1↓, M2↑, TNF-α↓,IL-6↓,IL-1β↓,IL-10↑,TGF-β↑,VEGF↑	Kamalipooya et al. (2024)
B. *In vivo* studies					
CNP-miR146a	miR146a conjugation	Diabetic mouse	Antioxidant, Angiogenesis	M2 polarization↑, IL-6↓	Zhou et al. (2022a)
CS-TA@CeO_2_ cryogel	Chitosan + Tannic acid	Infected full-thickness mouse wound	Antioxidant, Antibacterial, Angiogenesis, ECM remodeling	M2↑, fibroblast proliferation↑	Teng et al. (2022)
SBQCC/CNPs	Alginate + Chitosan	Full-thickness skin defect mouse	Angiogenesis, ECM remodeling	M2↑, CD31↑	Du et al. (2024)
MSN@Met-CeO_2_	Metformin-chelated mesoporous silica	Diabetic wound model	ROS scavenging, Hypoxia relief, Angiogenesis	Endothelial function↑	Li et al. (2024)
CIP-CeO_2_-PVs	PCL-b-PGA polymer vesicles + CeO_2_ NPs	Diabetic mouse infected wound	Antioxidant, Antibacterial, Wound healing	ROS↓, improved bacterial clearance	Wang et al. (2021)
CeO_2_-Y@ZIF-8@Gel	Cerium oxide-yttrium @ ZIF-8 @ hydrogel	Diabetic wound model/cells	Antioxidant, Anti-inflammatory	NLRP3↓, IL-1β↓, IL-18↓	He et al. (2024)

The symbols indicate changes in biological parameters (↑ increase, ↓ decrease).

#### 3.2.1 CeO_2_–antibiotic synergy in DFUs

The healing of DFUs, a common complication of diabetes, is often impaired by vascular damage and neutrophil dysfunction, which predispose wounds to bacterial infections. Conventional antibiotic therapies aim to inhibit bacterial growth by disrupting cell wall synthesis, compromising membrane integrity, or damaging bacterial genetic material. However, these treatments frequently fail, particularly in the presence of bacterial biofilms, and may ultimately necessitate amputation when infections become persistent or resistant (Li et al., 2023). Biofilms—dense, self-produced polymeric matrices—shield bacteria from host immune defenses and reduce antibiotic efficacy. Even systemic antibiotic therapy often cannot eradicate these sessile communities, contributing to chronic infection and delayed wound healing (Afonso et al., 2021).

To overcome these challenges, a synergistic approach combining antibiotics with CeO_2_ nanoparticles has been developed. Poly (ε-caprolactone)-block-poly (glutamic acid) polymer vesicles (PCL-b-PGA PVs) were functionalized *in situ* with ciprofloxacin (CIP) and stably decorated with CeO_2_ nanoparticles. At a relatively low cerium concentration of 1.25 μg/mL, these CIP-loaded, CeO_2_-adorned PVs (CIP-CeO_2_-PVs) suppressed superoxide radicals by approximately 50% through high superoxide dismutase (SOD)-mimicking activity. In normal L02 cells, CIP-CeO_2_-PVs provided optimal protection against oxidative damage at cerium levels of 5–20 μg/mL. Moreover, the combination enhanced antibacterial efficacy, allowing for a 25%–50% reduction in CIP dosage compared to free drug administration.

**FIGURE 4:**
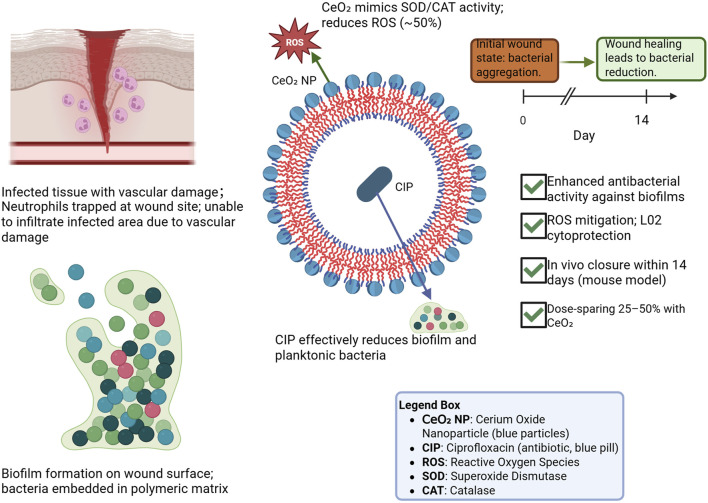
CeO_2_–ciprofloxacin synergy enhances antibacterial efficacy and wound healing in diabetic foot ulcers.

The therapeutic potential of CIP-CeO_2_-PVs was demonstrated *in vivo* using a diabetic mouse model, where treatment significantly accelerated the healing of infected wounds within 14 days (Wang et al., 2021). These findings indicate that the combined antioxidant and antibacterial activities of CeO_2_ nanoparticles synergize with antibiotics, outperforming either intervention alone (Figure 4). While this strategy represents a promising advancement in DFU management, further studies are needed to validate its generalizability and optimize dosing, toxicity profiles, and bioavailability.

#### 3.2.2 Chronic inflammation regulation and immunomodulation

**FIGURE 5:**
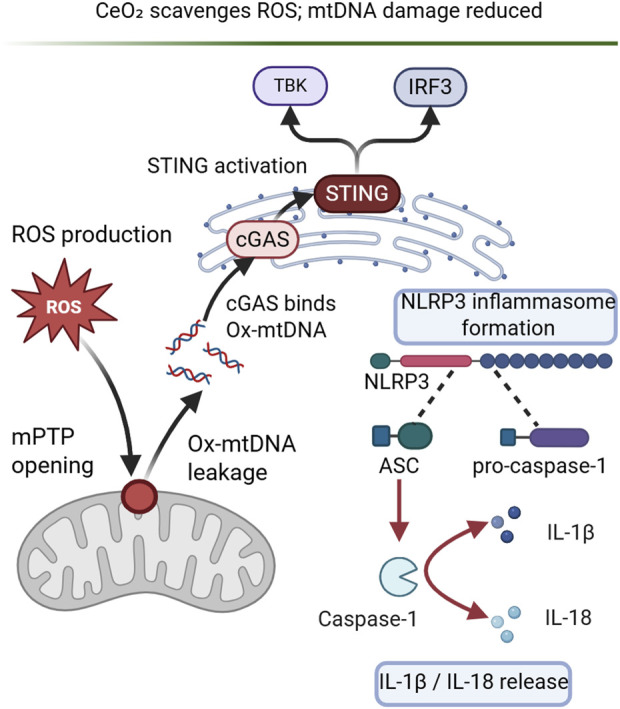
CeO_2_-Y@ZIF-8@Gel modulates the cGAS–STING–NLRP3 pathway in diabetic wound inflammation.

Activation of the NOD-like receptor protein 3 (NLRP3) inflammatory pathway hinders the healing of diabetic wounds. Shan He et al. developed a novel nano-enzyme-modified hydrogel (cerium oxide-yttrium@zeolitic imidazolate framework-8@hydrogel, abbreviated as CeO_2_-Y@ZIF-8@Gel) that can stop the activation of the NLRP3 inflammatory pathway. The cGAS-STING signaling pathway leading to NLRP3 inflammasome activation, with cerium oxide nanoparticles (CeO_2_ NP) playing a key role in reducing ROS and inhibiting NLRP3 activation (Figure 5). Cerium oxide (CeO_2_) may lessen mitochondrial damage by effectively scavenging excess intracellular reactive oxygen species (ROS) through the actions of catalase and superoxide dismutase (SOD).

Oxidatively damaged mitochondrial DNA (Ox-mtDNA), a damage-associated molecular pattern (DAMP), enters the cytoplasm through the mitochondrial permeability transition pore (mPTP). Ox-mtDNA production and leakage are reduced by CeO_2_-Y@ZIF-8@Gel through mitochondrial DNA (mtDNA) repair and ROS scavenging. Cyclic guanosine-adenylate synthase (cGAS) is bound and activated by Ox-mtDNA once it enters the cytoplasm. This activation triggers the stimulator of interferon genes (STING) pathway and encourages inflammatory reactions. By lowering Ox-mtDNA release, the cGAS-STING pathway is less activated, which prevents NLRP3 inflammatory vesicles from assembling and activating.

Inhibiting NLRP3 inflammatory vesicles also inhibits pro-caspase-1 aggregation and cleavage, which stops pro-inflammatory cytokines like interleukin-1 beta (IL-1β) and interleukin-18 (IL-18) from maturing and releasing. The CeO_2_-Y@ZIF-8@Gel-treated group exhibited a considerable decrease in NLRP3 expression, according to the results of Western blotting (WB). The enzyme-linked immunosorbent assay (ELISA) demonstrated a substantial decrease in IL-1β and IL-18 production incells pretreated with CeO_2_-Y@ZIF-8@Gel (He et al., 2024).

#### 3.2.3 Angiogenesis and ECM remodeling

Oxidative stress and chronic inflammation synergistically impair the expression of pro-angiogenic genes and proteins, constituting a major barrier to effective neovascularization in diabetic wounds. Therapeutic strategies targeting both oxidative and inflammatory pathways have shown potential in improving the wound microenvironment and promoting angiogenesis.

Dewberry et al. developed a cerium oxide nanoparticle–microRNA-146a complex (CNP-miR146a) that simultaneously attenuates oxidative stress and modulates inflammation via the NF-κB signaling pathway. MicroRNA-146a (miR146a) downregulates NF-κB activation by inhibiting key upstream mediators, including TNF receptor-associated factor 6 (TRAF6) and interleukin-1 receptor-associated kinase 1 (IRAK1). This downregulation suppresses the expression of pro-inflammatory cytokines such as IL-6 and IL-8, thereby reducing inflammation and enabling tissue repair. Concurrently, cerium oxide scavenges reactive oxygen species (ROS), mitigating oxidative damage. Treatment with CNP-miR146a resulted in enhanced expression of vascular endothelial growth factor (VEGF) and B-cell lymphoma 2 (Bcl-2), both of which are critical for early-stage angiogenesis and cell survival. *In vivo* results demonstrated improved wound architecture, increased collagen deposition, and enhanced dermal thickness. Fibroblast activity was also modulated in the presence of CNP-miR146a, with elevated miR146a levels observed in cultured fibroblasts, suggesting a potential role in ECM remodeling and cell motility. Although the precise molecular regulatory mechanisms remain to be fully elucidated, these findings highlight the multifaceted therapeutic effects of CNP-miR146a in diabetic wound healing (Dewberry et al., 2022).

Similarly, CS-TA@CeO_2_ cryogels, integrating CeO_2_ microcubes with chitosan and tannic acid (TA), provide antioxidant and antibacterial functions while supporting fibroblast proliferation and ECM deposition, resulting in enhanced angiogenesis and tissue regeneration in infected full-thickness wound models (Teng et al., 2022).^11^

**FIGURE 6:**
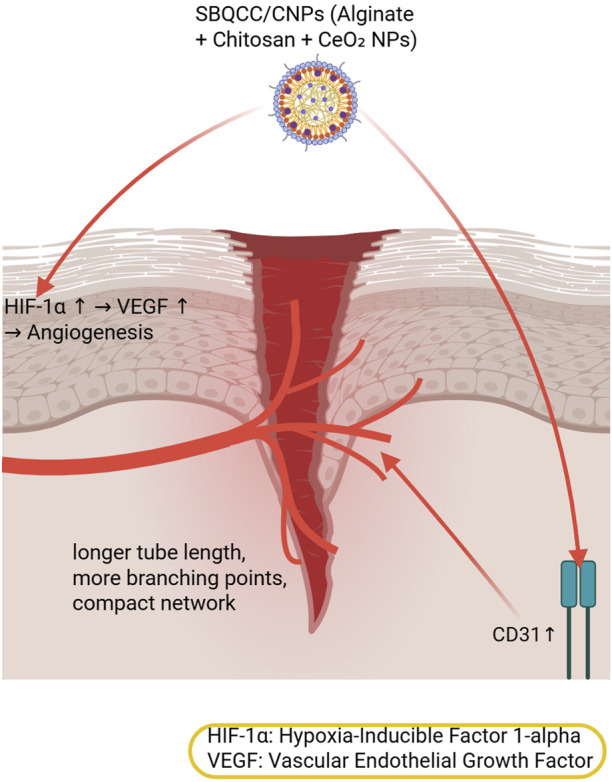
Mechanism of angiogenesis promotion by SBQCC/CNPs hemostatic powder.

Building on this multifunctional approach, Du et al. developed a self-gelatinizing hemostatic powder (SBQCC/CNPs) composed of alginate, chitosan, and bioactive CeO_2_ nanoparticles for hemorrhage control and wound healing. The CeO_2_ NPs preserved HIF-1α expression by modulating intracellular oxygen, thereby promoting angiogenesis. In a full-thickness skin defect mouse model, treatment with SBQCC/CNPs-0.1% significantly increased CD31 coverage (3.7% ± 0.3% vs. 1.4% ± 0.2% in controls), enhanced vascular network formation (longer tube length, more branching points, and greater compactness), and promoted M2 macrophage polarization, highlighting improved endothelial function and effective angiogenesis (Du et al., 2024). Figure 6 illustrates the mechanism of Du et al.’s SBQCC/CNPs hemostatic powder, showing how cerium oxide nanoparticles modulate intracellular oxygen to preserve HIF-1α and enhance CD31 expression, thereby promoting angiogenesis in a full-thickness diabetic wound model.

Collectively, these studies demonstrate the significant therapeutic potential of cerium oxide-based nanomaterials—such as CNP-miR146a, CS-TA@CeO_2_ cryogel, and SBQCC/CNPs powder—in addressing key pathological features of diabetic wounds. By modulating oxidative stress, reducing inflammation, and enhancing angiogenesis and ECM remodeling, these bioactive materials offer promising avenues for the development of advanced, multifunctional wound care platforms.

## 4 Inherent defects of uncoated CeO_2_ NPs

Uncoated CeO_2_ nanoparticles (NPs) suffer from intrinsic limitations, including physicochemical instability, poor biocompatibility, and functional singularity. High surface energy and redox lability cause aggregation, variable catalytic activity, and rapid *in vivo* clearance, while elevated doses may induce oxidative cytotoxicity. Additionally, bare NPs lack tissue selectivity, controlled release, and responsiveness to dynamic microenvironments, restricting therapeutic efficacy. Surface functionalization and structural engineering are therefore essential to enhance stability, biocompatibility, and multifunctionality for biomedical applications.

### 4.1 Physicochemical instability and biocompatibility

Uncoated cerium oxide nanoparticles (CeO_2_ NPs) have been shown to exhibit reasonable biocompatibility, even without surface functionalization. Several studies have demonstrated that CeO_2_ NPs can interact with biological systems without causing significant toxicity, primarily because their surface properties allow for efficient clearance and limited interaction with harmful reactive species (Monroy-Ramirez et al., 2025). However, the overall biocompatibility and effectiveness of CeO_2_ NPs *in vivo* depend heavily on factors such as size, aggregation behavior, and surface charge (Yadav et al., 2023; Ahmadzadeh et al., 2017).

The size of CeO_2_ NPs is a critical determinant of their behavior in biological environments. Smaller nanoparticles (∼5–10 nm) tend to be more biocompatible and exhibit better dispersion in physiological conditions. These particles can be cleared more efficiently by renal or hepatic excretion pathways due to their ability to interact less with plasma proteins, allowing them to avoid aggregation and achieve more effective elimination (Nosrati et al., 2023). On the other hand, larger particles tend to aggregate, which can lead to increased cytotoxicity and reduced efficacy in therapeutic applications (Yadav et al., 2023). These findings highlight the need for further research comparing the effects of uncoated and coated CeO_2_ NPs to understand how surface properties influence their biological interactions and therapeutic potential.

Moreover, uncoated CeO_2_ NPs are capable of forming highly stable water-based suspensions without surface coatings, which enhances their suitability for biomedical applications such as drug delivery and wound healing (Monroy-Ramirez et al., 2025; Allu et al., 2023). This inherent stability allows the nanoparticles to maintain dispersion and catalytic activity in biological environments, supporting therapeutic efficacy while minimizing toxicity (Walkey et al., 2015). Consequently, even in the absence of functional coatings, CeO_2_ NPs offer a significant advantage for applications that require both biocompatibility and redox activity.

Surface functionalization, however, can further improve the stability, biocompatibility, and functionality of CeO_2_ NPs. For instance, coating with biocompatible polymers or polyethylene glycol (PEG) can enhance the dispersion, reduce aggregation, and improve the interaction of CeO_2_ NPs with specific cell types, thereby increasing their therapeutic potential for chronic wound healing, including diabetic foot ulcers (Goujon et al., 2021). Local delivery via hydrogels or microneedle patches concentrates CeO_2_ NPs at the wound site, minimizing systemic exposure. Topical administration of up to 10 mg/kg is well tolerated, whereas intravenous doses above 1 mg/kg may induce systemic toxicity (Yuan et al., 2022; Erbay et al., 2025; Tian et al., 2024). These strategies allow sustained release, improve vascularization and fibroblast proliferation, and preserve bioactivity of co-delivered agents, optimizing safety and efficacy in DFU therapy.

In addition to physical instability, uncoated CeO_2_ NPs exhibit considerable chemical instability, particularly in redox-active biological environments. Under physiological pH (7.4), Ce^3+^ readily oxidizes to Ce^4+^ in the presence of oxidative agents such as hydrogen peroxide (H_2_O_2_), altering the Ce^3+^/Ce^4+^ ratio and thus their catalytic profile. While a higher Ce^3+^ content favors superoxide dismutase (SOD)-like activity, a lower ratio enhances catalase-like activity (Zhou D. et al., 2022). However, these redox transformations are often irreversible, especially under acidic conditions. For instance, Ce^4+^ species become unstable below pH 3.5, and Ce^3+^ cannot maintain its valence state below pH 6.0, leading to a decline in catalytic performance.

### 4.2 Clinical translation aspects

While cerium oxide nanoparticles (CeO_2_ NPs) exhibit great promise in biomedical applications, particularly in diabetic foot ulcer (DFU) therapy, their clinical translation faces several challenges. These challenges include scalability, regulatory requirements, reproducibility, and long-term biosafety. Addressing these factors is essential for ensuring the successful transition of CeO_2_ NPs from laboratory research to clinical applications.

One of the key barriers to the clinical application of CeO_2_ NPs is the scalability of their production. Currently, most CeO_2_ NP synthesis methods are optimized for laboratory-scale production, and scaling up these processes while maintaining particle consistency and high quality is a significant challenge. Factors such as synthesis cost, batch-to-batch variability, and the potential for nanoparticle aggregation during large-scale production need to be addressed. Strategies such as microfluidic-based synthesis, automated production lines, and optimization of green synthesis methods could offer potential solutions for large-scale, cost-effective manufacturing of CeO_2_ NPs without compromising their therapeutic efficacy (El-Saadony et al., 2024; Rajeshkumar and Naik, 2018).

The regulatory approval of CeO_2_ NPs for clinical use involves rigorous evaluation by regulatory bodies, such as the U.S. Food and Drug Administration (FDA), European Medicines Agency (EMA), and China National Medical Products Administration (NMPA). These agencies require comprehensive data on the safety, efficacy, and quality of nanomaterials (Csóka et al., 2021). For CeO_2_ NPs, this means conducting extensive preclinical studies, including toxicological assessments, biodistribution studies, and long-term safety evaluations. Regulatory agencies also require clinical trials to establish the safety and efficacy of CeO_2_ NPs in human subjects. As CeO_2_ NPs may exhibit unique biological interactions due to their redox activity, understanding these interactions is crucial for their clinical approval.

Another critical aspect of clinical translation is ensuring the reproducibility of CeO_2_ NP properties across different batches and production sites. Variations in particle size, surface charge, and Ce^3+^/Ce^4+^ ratios can impact their biological performance, including their antioxidant activity, biocompatibility, and therapeutic efficacy. Establishing standardized protocols for the synthesis, characterization, and quality control of CeO_2_ NPs is essential for ensuring consistent product performance. This includes developing reliable techniques for nanoparticle characterization, such as dynamic light scattering (DLS), transmission electron microscopy (TEM), and zeta potential analysis, to monitor their properties in real-time and across different production batches (Shende et al., 2015; Nikolaeva et al., 2021).

Although CeO_2_ NPs have shown excellent biocompatibility and low toxicity in short-term studies, their long-term biosafety remains a significant concern. The long-term accumulation of CeO_2_ NPs in organs, particularly the liver, kidneys, and spleen, must be carefully evaluated to prevent potential toxicity. Additionally, the interaction of CeO_2_ NPs with biological systems, including immune responses, inflammatory reactions, and oxidative stress, needs to be further understood. Long-term animal studies and clinical trials should focus on evaluating the chronic exposure effects of CeO_2_ NPs, assessing potential organ-specific toxicity, and determining their bioaccumulation and clearance pathways (Casals et al., 2020; Attia et al., 2022). Strategies such as surface functionalization with biocompatible coatings or the design of degradable CeO_2_ NPs may reduce the risk of long-term accumulation and enhance their safety profile.

In addition to the technical and regulatory hurdles, there are also clinical application challenges associated with the use of CeO_2_ NPs in wound healing therapies. The complexity of DFUs, which involve multiple factors such as inflammation, infection, and poor circulation, requires a multifaceted approach to therapy (Armstrong et al., 2023). CeO_2_ NPs’ ability to modulate oxidative stress and inflammation makes them a promising candidate for DFU treatment, but their use in combination with other therapeutic agents, such as antibiotics, growth factors, and stem cells, should be explored to maximize their efficacy. Furthermore, the development of targeted delivery systems, such as hydrogel-based patches, microneedles, or nanoparticle conjugates, could improve the localized application of CeO_2_ NPs to the wound site while minimizing systemic exposure and potential toxicity (Augustine et al., 2021; Dewberry et al., 2022; Tian et al., 2024).

### 4.3 Functional singularity of uncoated CeO_2_ NPs

In systemic applications, uncoated nanoparticles face significant limitations, including poor tissue selectivity, lack of controlled drug release, and risk of off-target effects. These particles cannot often distinguish between healthy and pathological tissues, increasing the risk of systemic toxicity. Furthermore, unmodified NPs are unable to respond to the dynamically evolving microenvironment of damaged tissues, such as wounds or inflammatory lesions. Their typical release profile involves an abrupt and non-specific drug release, which cannot be modulated in response to localized physiological cues, thereby compromising therapeutic efficacy and increasing the potential for adverse effects.

Similarly, Salarieh et al. fabricated cubosomal nanostructures incorporating biologically derived CeO_2_ NPs (produced by *Lactobacillus* acidophilus) for dual drug loading with glatiramer acetate and carboxymethylcellulose (CMC) as a coating agent. *In vitro* release assays performed in phosphate-buffered saline (PBS, pH 7.4) at 37 °C ± 2 °C under continuous stirring revealed an initial burst release followed by sustained, gradual drug release across multiple formulations. This controlled release profile underscores the potential of cubosome-based systems to maintain therapeutic drug concentrations over extended periods (Salarieh et al., 2023).

Collectively, these engineered systems illustrate how thoughtful surface and structural design can overcome biological barriers, modulate release kinetics, and align nanoparticle activity with the complex demands of wound microenvironments, offering clear advantages over unmodified particles.

### 4.4 Future directions in CeO_2_ NPs functionalization for wound healing and drug delivery

In conclusion, uncoated CeO_2_ nanoparticles hold considerable promise for biomedical applications owing to their inherent biocompatibility, catalytic activity, and stability across diverse biological environments. Nonetheless, their therapeutic efficacy—particularly in chronic wound healing and drug delivery—is often constrained by issues such as functional singularity, aggregation, and limited tissue selectivity. Surface functionalization and structural modifications present viable strategies to enhance nanoparticle stability, biocompatibility, and multifunctionality, thereby improving their performance in targeted therapies.

Building on the synthesis and surface optimization strategies outlined in Section 3.1, future research should focus on elucidating the interactions between CeO_2_ NPs and endothelial progenitor cells (EPCs) or endothelial colony-forming cells (ECFCs). A deeper understanding of how CeO_2_ NPs influence EPC/ECFC survival, angiogenic potential, and functional behavior could unlock new avenues for promoting vascular repair in diabetic foot ulcers (DFUs) and other chronic wounds. Furthermore, advanced surface functionalization techniques and controlled-release systems will be essential to maximize therapeutic efficacy while maintaining biocompatibility. Precision delivery platforms, such as hydrogel-based matrices or microneedle systems, can further enhance local retention and minimize systemic exposure, enabling safe and effective multifunctional therapies for chronic wound management.

## 5 Evolution of coating technologies

The development of nanoparticle coating strategies has undergone three major stages, transitioning from simple monolayer adsorption to multifunctional, stimuli-responsive hybrid systems. Each stage reflects a response to the growing complexity of biomedical applications and the need for enhanced stability, specificity, and therapeutic functionality of cerium oxide nanoparticles (CeO_2_ NPs).

Stage I involved physically adsorbed monomolecular coatings, such as citric acid or polyvinylpyrrolidone (PVP), which were primarily designed to reduce particle aggregation and improve colloidal dispersion. While these coatings were straightforward to apply, they lacked structural robustness, were susceptible to desorption, and offered limited functional versatility.

To overcome these constraints, researchers introduced more bioactive surface modifications. For example, Yuhua Sun et al. employed a polydopamine (pDA) coating strategy to functionalize nanohydroxyapatite (nano-HA) for improved osteogenic potential. Dopamine was self-polymerized under alkaline conditions (pH 8.5) and elevated temperature (60 °C), forming a pDA layer with abundant reactive groups. While this coating demonstrated favorable bioactivity, challenges remained. The synthesis required stringent pH and thermal control, and incomplete removal of physically adsorbed molecules after dialysis could compromise purity. Moreover, high-temperature processes (∼900 °C) necessary for thermogravimetric stability risked altering the underlying nano-HA structure. Variability in coating uniformity further limited reproducibility, potentially affecting peptide conjugation and downstream biological outcomes (Sun et al., 2013).

In another example, Zahra Sharifalhoseini et al. applied a silica shell onto ZnO NPs using tetraethoxysilane (TEOS) hydrolysis in an ethanol–ammonia system. The silica layer significantly improved chemical stability, reduced agglomeration, and enhanced compatibility for composite integration. However, coating uniformity remained a concern, particularly on complex geometries. Irregular TEOS addition could result in incomplete or over-deposition, and a mismatch in thermal expansion between core and shell materials, introducing risks of delamination or cracking. Additional pretreatment or surface modification steps were often required to ensure adequate adhesion (Sharifalhoseini and Entezari, 2015).

Stage II saw the emergence of covalently bonded functional coatings that improved stability and enabled molecular conjugation. Covalent grafting of polyethylene glycol (PEG), aminosilanes, and other reactive moieties became common. Xiangsheng Liu et al. developed core–shell GNP@pDA nanostructures by anchoring pDA layers onto gold nanoparticles (GNPs) via π–π stacking and covalent interactions. The resulting coating provided high biocompatibility, functional group abundance (quinones, hydroxyls, amines), and suitability for drug delivery and imaging. However, the very abundance of active sites could lead to non-specific adsorption, limiting selectivity. Moreover, the thickness and consistency of pDA layers were highly sensitive to environmental factors such as dopamine concentration, pH, and temperature, necessitating careful process control (Liu et al., 2013).

Phase III consists of multifunctional hybridized coatings (2021–present) that facilitate intelligent response and synergistic therapy. Silica-polyethylene glycol (SiO_2_–PEG) double coating is a prime instance of an inorganic and organic hybridization that reduces toxicity and prolongs half-life. As a multifunctional hybridized coating, poly-3-hydroxybutyrate-chitosan (PHB–Cs)/multi-walled carbon nanotubes (MWCNTs) combines the benefits of several material compositions, such as polymers (PHB, chitosan) and nanomaterials (MWCNTs), to create a composite system. It enhanced the nano bioactive glass–nano titanium dioxide (nBG–nTiO_2_) scaffolds’ mechanical characteristics (such as a 30-fold increase in compressive strength) and biological activities (such as MG-63 cell survival and proliferation promotion and alkaline phosphatase secretion enhancement), making them appropriate for bone tissue engineering applications (Rajeshkumar and Naik, 2018).

Multifunctionality is also exemplified by poly (PEGMA-r-phosphomer) coatings, where PEG brushes confer anti-adhesive and antibacterial properties, while phosphomer groups support cell attachment. This dual-function design resolves the typical trade-off between bacterial resistance and tissue integration, allowing for both infection control and improved osteocompatibility (Cui et al., 2018).

The most recent trend involves stimuli-responsive coatings, particularly pH-sensitive systems. For instance, ionic cross-linking of chitosan (CS) with carboxymethyl chitosan (CMCS) yields PE@CS/CMCS–NPs, which swell and release their payload in acidic environments (e.g., pH 6.5) characteristic of chronic wounds. Such coatings enable targeted and controlled drug release, aligning therapeutic output with microenvironmental cues. When applied to CeO_2_ NPs, these responsive systems provide a promising platform for treating diabetic foot ulcers (DFUs), where dynamic control over inflammation, infection, and tissue regeneration is critical (Zhou Y. et al., 2022).

Through this progressive evolution—from single-purpose physical coatings to intelligent, multifunctional, and responsive architectures—coating technologies have addressed longstanding limitations of CeO_2_ NPs, including poor colloidal stability, off-target toxicity, and lack of therapeutic specificity. These advances not only enhance the translational potential of CeO_2_-based platforms but also redefine their role as active participants in wound healing rather than passive carriers.

## 6 Discussion

This review systematically evaluates the therapeutic potential of engineered cerium oxide nanoparticles (CeO_2_ NPs) in diabetic foot ulcer (DFU) management, emphasizing their multifunctionality in counteracting oxidative stress, chronic inflammation, microbial biofilms, impaired angiogenesis, and extracellular matrix (ECM) dysregulation. Advances in surface engineering—through polymeric, biomolecular, and hybrid coatings—have significantly improved the biocompatibility, catalytic efficiency, and stability of CeO_2_ NPs. Notable nanoformulations such as MSN@Met-CeO_2_ and CS-TA@CeO_2_ demonstrated favorable preclinical outcomes, supporting translational potential in chronic wound treatment (Zhao C. et al., 2024; Teng et al., 2022).

Despite promising *in vitro* antioxidant profiles, with some reports indicating over 80% reactive oxygen species (ROS) clearance, CeO_2_ NPs often exhibit reduced catalytic activity *in vivo* due to the complex physiological environment. Protein corona formation, pH-sensitive redox cycling, and diverse biological interactions diminish functional persistence. For example, Sol Guerra-Ojeda et al. observed only 20%–30% ROS reduction in murine liver tissue despite strong *in vitro* performance (Guerra-Ojeda et al., 2022) This highlights the critical need for coatings that stabilize the Ce^3+^/Ce^4+^ ratio under dynamic *in vivo* conditions. Synergistic platforms, such as ciprofloxacin-loaded cerium–polymer vesicles (CIP-Ceria-PVs), demonstrate that combination strategies can lower antibiotic doses while maintaining antimicrobial efficacy, offering a viable approach to counteract resistance (Wang et al., 2021).

Several limitations remain unresolved. CeO_2_ NPs exhibit heterogeneous biodistribution, frequently accumulating in off-target organs like the liver and spleen. Coatings such as PLGA enhance pharmacokinetics but may degrade into lactic acid, transiently acidifying the microenvironment and potentially impairing fibroblast proliferation and ECM remodeling. Large-scale production of hybrid-coated NPs (e.g., SiO_2_–PEG) faces technical and economic barriers, with costs increasing five-to ten-fold and requiring specialized synthesis methods. Furthermore, stimuli-responsive coatings often rely on *in vitro* activation thresholds (e.g., pH 6.5) that may not accurately represent wound physiology, and the absence of real-time *in situ* monitoring in humans limits clinical predictability.

Nevertheless, CeO_2_ NPs, when delivered via targeted and intelligent systems such as GelMA-based microneedle arrays or pH-/ROS-responsive hydrogels, offer coordinated therapeutic effects on multiple DFU pathophysiological components. Constructs including exosome-loaded CeO_2_ microneedle patches exemplify next-generation approaches, integrating molecular precision with real-time responsiveness in line with personalized and regenerative medicine paradigms (Yuan et al., 2022; Erbay et al., 2025; Tian et al., 2024). Future research should prioritize subtype-specific formulations for ischemic versus neuropathic DFUs, standardization of toxicity evaluation frameworks including reproductive and long-term biodistribution studies, and data-driven optimization of nanoparticle design through machine learning. Scalable, green, bioinspired synthesis methods and biofeedback-responsive platforms capable of adjusting release kinetics to biomarkers (e.g., interleukin levels, tissue pH, glucose) are essential for clinical translation. Large-scale, multicenter clinical trials remain critical to establish dosing, safety, and efficacy under chronic or high-dose exposure conditions.

Engineered CeO_2_ NPs thus represent a multifunctional and adaptable platform for DFU therapy, simultaneously addressing redox imbalance, infection, inflammation, and impaired regeneration. Their integration with advanced coatings and smart delivery systems positions them as next-generation regenerative therapeutics. Successful clinical adoption will depend on overcoming challenges related to safety, scalable production, and real-time therapeutic control, ultimately bridging nanotechnology with translational medicine to unlock the full clinical potential of CeO_2_ NPs in diabetic wound care.

## 7 Conclusion

Cerium oxide nanoparticles (CeO_2_ NPs) offer considerable promise in addressing key challenges associated with diabetic foot ulcers (DFUs), including oxidative stress, inflammation, and impaired wound healing. Their biocompatibility, stability, and therapeutic efficacy have been significantly enhanced through various coating techniques, further improving their potential for clinical application. However, challenges such as dose-dependent toxicity, scalability in manufacturing, and the need for uniform safety protocols still pose significant hurdles.

To facilitate their broader clinical adoption, future research should focus on several strategic areas. Developing smart-responsive coatings that can dynamically adjust to the changing conditions of the wound microenvironment would optimize the therapeutic efficacy of CeO_2_ NPs. Additionally, integrating CeO_2_ NPs with biologics or gene therapies could amplify their regenerative potential, addressing the multifaceted nature of DFU healing. Furthermore, the advent of personalized nanomedicine approaches, tailored to the unique needs of different DFU subtypes, could ensure more effective and targeted treatment.

By continuing to refine the design of CeO_2_ NPs, enhancing their safety profiles, and integrating them with cutting-edge therapeutic technologies, CeO_2_ NPs have the potential to revolutionize the treatment of chronic wounds, particularly in diabetic patients, and transform the landscape of wound care.

## Glossary

DFU: Diabetic foot ulcer
**CeO**_**2**_ **NPs**: Cerium oxide nanoparticles
ROS: Reactive oxygen species
ECM: Extracellular matrix
AGEs: Advanced glycation end-products
**MSN@Met-CeO**_**2**_: Mesoporous Silica Nanoparticle loaded with Metformin and Cerium Dioxide
DRP1: Dynamin-related protein one
PVs: Polymer vesicles
PLGA: Poly(lactic-co-glycolic acid)
**SiO**_**2**_: Silicon dioxide
PEG: Polyethylene glycol
GelMA: Gelatin methacryloyl
PEGDA: Poly(ethylene glycol) diacrylate
Tau: Taurine
CTH@MN: CeO_2_@Tau hydrogel microneedle patch
Exos: Exosomes
HIF-1α: Hypoxia-inducible factor 1-alpha
SOD: Superoxide dismutase
CAT: Catalase
GPx: Glutathione peroxidase
**CS-TA@CeO**_**2**_: Chitosan–tannic acid–cerium oxide cryogel
CIP-Ceria-PVs: Ciprofloxacin-loaded cerium-decorated polymer vesicles
pH: Potential of hydrogen
ML: Machine learning
NPs: Nanoparticles
SBQCC/CNPs: Sodium alginate-benzeneboronic acid/Quaternized chitosan-catechol with Cerium oxide nanoparticles
MSC: Mesenchymal stem cell
EPCs: Endothelial progenitor cells
ECFCs: Endothelial colony-forming cells
