# Correction: Cerium oxide nanoparticles for enhanced healing of diabetic foot ulcers

**DOI:** 10.3389/fbioe.2026.1787328

**Published:** 2026-02-06

**Authors:** Ziyue Fu, Ling Chen, Lingjiang Mo, Yuefu Zhan, Liheng Ma

**Affiliations:** 1 The First Affiliated Hospital of Guangdong Pharmaceutical University, Guangzhou, China; 2 The Third People’s Hospital of Longgang District Shenzhen, Shenzhen, China

**Keywords:** cerium oxide nanoparticles, diabetic foot ulcers, coating strategies, antioxidant activity, anti-inflammatory, biocompatibility, angiogenesis, extracellular matrix remodeling

The order of authors in the **Author list** of the published paper was erroneously given as:

Ziyue Fu^1^, Ling Chen^1^, Lingjiang Mo^1^, Liheng Ma^1^* and Yuefu Zhan^2^*

The correct **Author list** reads:

Ziyue Fu^1^, Ling Chen^1^, Lingjiang Mo^1^, Yuefu Zhan^2^ *, Liheng Ma^1^ *

The **Author contributions statement** was erroneously given as “ZF: Writing – original draft, Writing – review and editing. LC: Writing – original draft, Writing – review and editing. LMo: Writing – review and editing. LMa: Writing – review and editing. YZ: Writing – review and editing.” The correct **Author contributions statement** is “ZF: Writing – original draft, Writing – review and editing. LC: Writing – original draft, Writing – review and editing. LMo: Writing – review and editing. YZ: Writing – review and editing. LMa: Writing – review and editing.”

Affiliation “2The Third People’s Hospital of Longgang District Shenzhen, Shenzhen, China” was erroneously given as “2Guangdong Pharmaceutical University, Guangzhou, China”.

The original article has been updated.

